# Market access and value-based pricing of digital health applications in Germany

**DOI:** 10.1186/s12962-022-00359-y

**Published:** 2022-06-13

**Authors:** Daniel Gensorowsky, Julian Witte, Manuel Batram, Wolfgang Greiner

**Affiliations:** 1 School of Public Health, Department of Health Economics and Health Care Management, Bielefeld University, P.O. Box 10 01 31, D-33501 Bielefeld, Germany; 2 Vandage GmbH, Detmolder Straße 30, D-33604 Bielefeld, Germany

**Keywords:** Digital health applications, DiGA, eHealth, Statutory health insurance, Market access, Value-based pricing, Fast-track process

## Abstract

In December 2019, the Digital Health Care Act (“Digitale-Versorgung-Gesetz”) introduced a general entitlement to the provision and reimbursement of digital health applications (DiGA) for insured persons in the German statutory health insurance. As establishing a new digital service area within the solidarity-based insurance system implies several administrative and regulatory challenges, this paper aims to describe the legal framework for DiGA market access and pricing as well as the status quo of the DiGA market. Furthermore, we provide a basic approach to deriving value-based DiGA prices.

To become eligible for reimbursement, the Federal Institute for Drugs and Medical Devices evaluates the compliance of a DiGA with general requirements (e.g., safety and data protection) and its positive healthcare effects (i.e., medical benefit or improvements of care structure and processes) in a fast-track process. Manufacturers may provide evidence for the benefits of their DiGA either directly with the application for the fast-track process or generate it during a trial phase that includes temporary reimbursement. After one year of \]reimbursement, the freely-set manufacturer price is replaced by a price negotiated between the National Association of Statutory Health Insurance Funds and the manufacturer. By February 2022, 30 DiGA had successfully completed the fast-track process. 73% make use of the trial phase and have not yet proven their benefit. Given this dynamic growth of the DiGA market and the low minimum evidence standards, fair pricing remains the central point of contention. The regulatory framework makes the patient-relevant benefits of a DiGA a pricing criterion to be considered in particular. Yet, it does not indicate how the benefits of a DiGA should be translated into a reasonable price. Our evidence-based approach to value-based DiGA pricing approximates the SHI’s willingness to pay by the average cost-effectiveness of one or more established therapy in a field of indication and furthermore considers the positive healthcare effects of a DiGA.

The proposed approach can be fitted into DiGA pricing processes under the given regulatory framework and can provide objective guidance for price negotiations. However, it is only one piece of the pricing puzzle, and numerous methodological and procedural issues related to DiGA pricing are still open. Thus, it remains to be seen to what extent DiGA prices will follow the premise of value-based pricing.

## Background

The low level of digitalization in the German healthcare system compared to other countries has been documented in various studies in recent years [1, 2]. The reasons for this disparity are manifold. Data privacy issues and a lack of support for the joint digitalization project by the various players in the self-governing healthcare system are considered to be primary causes [3]. Under these conditions, the nationwide introduction of electronic health records alone took over 15 years. However, several efforts were made in the previous legislative period (2017–2021) to catch up with the existing development backlog. A pioneering step, for Germany and internationally, toward comprehensive digitally supported patient care took place at the end of 2019. With the Digital Healthcare Act (“Digitale-Versorgung-Gesetz”, DVG), the German legislature introduced a general entitlement to the provision and reimbursement of digital health applications (“Digitale Gesundheitsanwendungen”, DiGA) for the approximately 73 million insured persons in the statutory health insurance (SHI)—thereby creating nothing less than an entirely new, digital service area in SHI. Colloquially referred to as “prescription apps”, DiGA are low-risk medical products (risk class I or IIa according to Medical Device Regulation [MDR] or, under the transitional provisions of the MDR, according to Medical Device Directive [MDD]) whose primary function is based on digital technologies. The first reimbursable DiGA entered the healthcare market in October 2020. To date (as of 02/28/2022), the number of available DiGA has increased to 30. As Smartphone applications or browser-based web applications and across various indications, they support the detection, monitoring, treatment, or alleviation of diseases or the detection, treatment, alleviation, or compensation of injuries or disabilities. Applications from the field of primary prevention are not covered by the DiGA definition.

Establishing a new digital service area within the solidarity-based SHI system is accompanied by numerous administrative and regulatory challenges. Therefore, this paper aims to describe the regulatory framework for DiGA market access and pricing and the current state of the DiGA market. Furthermore, we provide an approach to deriving a reasonable price of a DiGA based on its demonstrated positive effects on healthcare.

## Market access of DiGA

General regulations for DiGA were set up in Book V of the German Social Code (“Sozialgesetzbuch V”, SGB V). Provisions on market access and pricing have been further specified by the Digital Health Applications Regulation (“Digitale Gesundheitsanwendungen-Verordnung”, DiGAV) as well as the framework agreement between the National Association of Statutory Health Insurance Funds (“GKV-Spitzenverband”, GKV-SV) and the umbrella organizations of manufacturers on the standards for contracts on negotiated prices according to Sect. 134 (4) and (5) SGB V [4]. These legal guidelines differ significantly from other nationally and internationally established benefit assessment and market access procedures, particularly regarding the required level of evidence, the speed of the assessment process and the rules governing pricing decisions.

Eligibility for reimbursement of a DiGA depends on compliance with general requirements regarding safety, functional capability, quality, data security, and data protection. In addition, manufacturers must provide evidence of the “positive healthcare effects” (“positive Versorgungseffekte”, pVE) of their DiGA versus “non-application” based on a comparative study. Generally, Sect. 139e (9) Sentence 2 SGB V stipulates that the evidence requirements for the proof of pVE must follow the principles of evidence-based medicine. However, with retrospective, comparative studies (incl. pre-post comparisons) as the first choice, the DiGAV formally defines a rather low standard for DiGA benefit assessment compared with requirements for other healthcare services. In contrast, higher-quality prospective studies such as randomized controlled trials (RCT) are considered only as a possible alternative to retrospective studies. The “non-application” comparator required has to consist of a care modality which does not use the DiGA under investigation. An official determination of an appropriate comparator therapy, such as in the course of the early benefit assessment for new drugs (the so-called AMNOG procedure), is not the subject of evaluation process. However, the regulatory framework requires that the comparator must reflect the reality of care in the specific indication. Thus, it can also consist of a usual care mix instead of one clearly defined standard therapy. The pVE themselves represent a conceptual neologism. The term is broadly defined and includes both the medical benefit of a DiGA as well as so-called “patient-relevant improvements of structure and processes” (“patientenrelevante Struktur- und Verfahrensverbesserungen”, pSVV). Medical benefits comprise, in particular, patient-relevant effects concerning improving health status, shortening the duration of disease, prolonging survival, or improving quality of life. PSVV, on the other hand, aim to support patients’ healthcare activities or integrate processes between patients and healthcare providers. According to Sect. 8 (3) DiGAV, they particularly consider aspects such as:

- Coordination of treatment procedures.
- Alignment of treatment with guidelines and recognized standards.
- Adherence.
- Facilitating access to care.
- Patient safety.
- Health literacy.
- Patient sovereignty.
- Coping with illness-related difficulties in everyday life.
- Reduction of therapy-related efforts and strains for patients and their relatives.

**Fig. 1:**
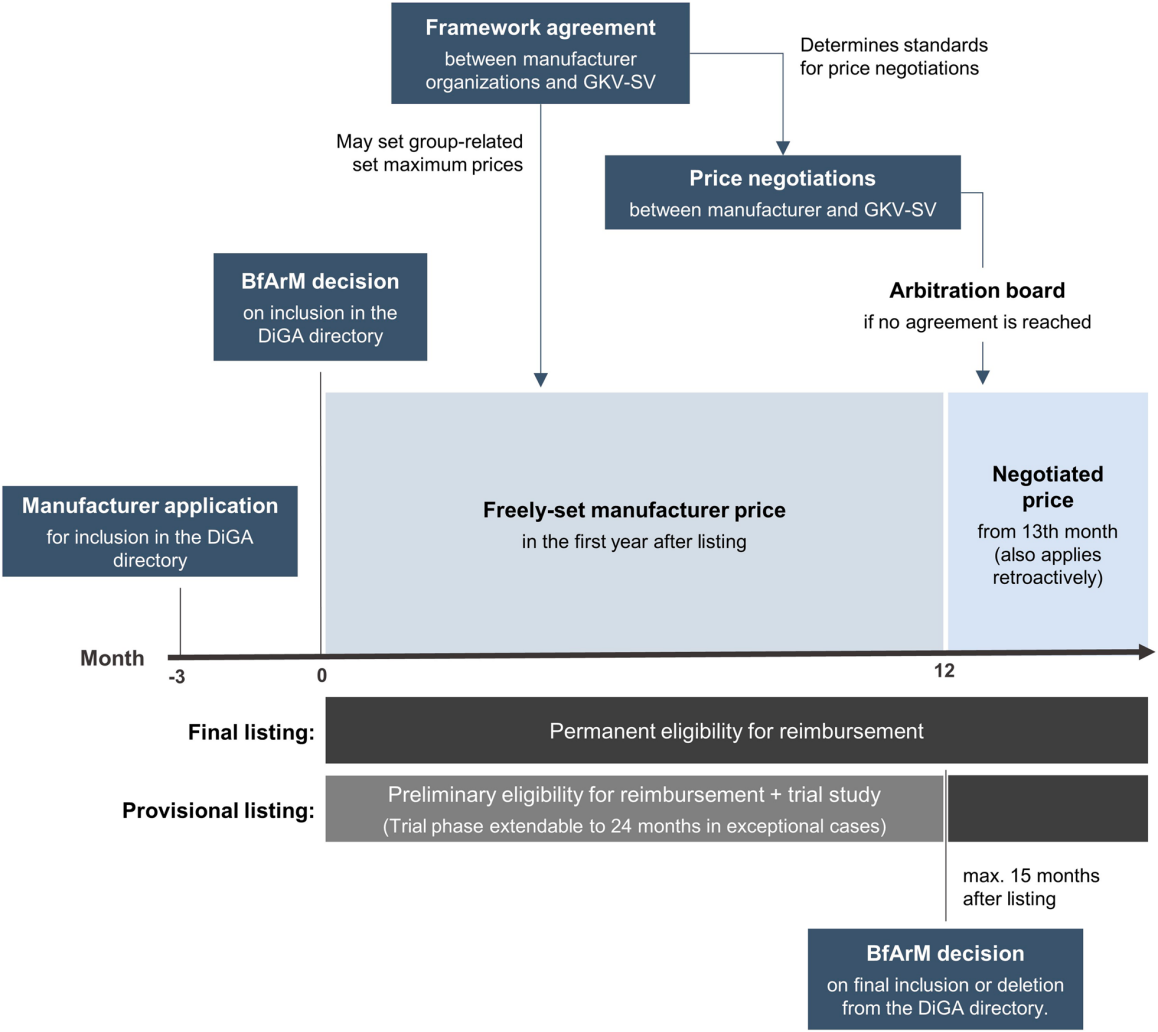
Schematic overview of the evaluation and pricing process (“fast-track process”) for DiGA

To assess whether a DiGA meets the requirements for reimbursement, an application and evaluation procedure has been established at the Federal Institute for Drugs and Medical Devices (“Bundesinstitut für Arzneimittel und Medizinprodukte”, BfArM). Due to the short duration of procedural steps, this is also referred to as the “fast-track process”. The BfArM provides detailed instructions on the processes and requirements in guidance available on its website [5]. A schematic overview of the fast-track process is given in Fig. 1. At the end of the evaluation process, if successful, the corresponding DiGA is included in the DiGA directory of the BfArM. The online directory contains relevant, comprehensive information on all reimbursable DiGA and is accessible for patients, healthcare providers, and other interested parties [6]. The decision of the BfArM on the approval is made within three months after the manufacturer has submitted the complete application. A clock stop in case of additional requests by the BfArM is not possible. Manufacturers can provide evidence for the pVE of their DiGA either with the initial application for inclusion in the directory or, if a study is not yet available, generate this evidence during a provisional listing. The latter is intended as a trial phase during which DiGA manufacturers collect the necessary study data concerning pVE. The trial period is regularly limited to one year but can be extended to a maximum of two years in exceptional cases. At the end of this period, the BfArM makes its determination on the existence of pVE and thus a decision on the final inclusion of the DiGA in the DiGA directory. When applying for provisional listing, the manufacturer must plausibly demonstrate the potential pVE of their DiGA and that the planned trial will be able to generate sufficient evidence on pVE. For this purpose, the manufacturer submits to the BfArM the results of a systemic data evaluation of the DiGA and a scientific evaluation concept for the trial planned to prove the pVE. DiGA for which the evidence for pVE is already provided with the initial application will be permanently included in the DiGA directory directly after a positive decision by the BfArM.

All DiGA in the directory are reimbursable by the SHI, and this applies regardless of whether the listing is already permanent or initially only provisional. In the event of illness, insured persons in the SHI have two options for getting access to a suitable DiGA: a prescription from their treating physician or therapist or a direct application to their health insurance fund. In both cases, the diagnosis addressed by the respective DiGA must be documented for the patient, and any contraindications must be excluded.

At the beginning of October 2020, the DiGA directory went online with the first two DiGA – the tinnitus app “Kalmeda” (provisional listing) and the web application “velibra” (final listing) for treatment support in anxiety disorders. Three more DiGA successfully completed the BfArM review process and became reimbursable in the same month. To date (as of 02/28/2022), 123 applications for a listing in the DiGA directory have been submitted—92 for a provisional and 31 for a final listing [7]. Of these, 30 received a positive decision from the BfArM, which led to inclusion in the DiGA directory. On average, the new digital care sector is thus growing continuously by two new DiGA per month. Another 24 applications are currently being processed; eight were rejected by the BfArM, and 61 were withdrawn by the applying manufacturer. Detailed information on unsuccessful applications is not available. However, the BfArM explains the high proportion of withdrawals by the fact that many manufacturers need additional time to prepare all the documents required for the assessment [8]. In addition, lack of compliance with the evidence requirements is a key reason for withdrawn or negatively adjudicated applications.

The 30 applications currently listed in the DiGA directory aim to support patients with a wide range of diseases – from obesity to cancer to stroke – in their treatment and/or management of therapy (see Table 1). 73% (22/30) of these use or used the provisional listing option to generate the evidence of medical benefit or pSVV necessary for a final listing in the DiGA directory. Two applications—“Kalmeda” and “Vivira”—have already successfully completed their trial phase and have thus been finally listed. Thus, 20 applications are currently reimbursable to the SHI system without final proof of benefit. The ten listed DiGA have proven their pVE in at least one RCT and thus exceeded the low minimum evidence standard required for a listing. Study sample sizes vary considerably for the various DiGA listed so far—from 56 participants (“somnio”; insomnia) to 1013 participants (“deprexis”; depression). Most studies compared usual care extended by the respective DiGA with a waiting list control group receiving usual care alone (rather unspecified in most cases). In the “HelloBetter Diabetes and Depression” study, usual care in the control group was supplemented by an online psychoeducational program for the treatment of unipolar depression. Although both a medical benefit and pSVV alone can be used to demonstrate a pVE, the ten permanently listed DiGA base their pVE proof on at least a medical benefit in terms of reductions in disease-related symptoms. However, two of these DiGA have also proven pSVV in terms of reduced treatment-related costs and burdens for patients and family members (“velibra”, anxiety disorders) or increased patient sovereignty (“vorvida”, alcohol use disorders). Reductions in treatment-related costs and burdens are based on improvements in overall psychological distress, measured via the validated Brief Symptom Inventory (BSI) [9]. Patient sovereignty was operationalized as self-efficacy among patients with alcohol use disorders, which was measured via the validated Alcohol Abstinence Self-Efficacy Questionnaire (AASE-G) [10]. A detailed overview of the addressed pVE of all applications listed in the DiGA directory is given in Table 2.

**Table 1:** General characteristics and prices of DiGA listed in the DiGA directory (as of 02/28/2022).

DiGA-Name	Indication(s)	Primary intervention principle(s)	Date of listing	Listing status (initial / current)	pVE^a^	Application type	Prescription period	MP^b^ per prescription	NP^c^ per prescription (discount on MP)
Kalmeda	Tinnitus	CBT	09/25/2020	P / F	MB	App	90 days	203.97 €	–
velibra	Generalized anxiety Disorder, Panic disorder (with/without agoraphobia), Social phobias	CBT	10/01/2020	F	MB, PSVV	Browser	90 days	476.00 €	–
somnio	Insomnia	CBT	10/22/2020	F	MB	App, Browser	90 days	464.00 €	224.99 € (–52 %)
Vivira	Back pain	Physiotherapy	10/22/2020	P / F	MB	App	90 days	239.96 €	–
zanadio	Obesity	multimodal obesity therapy	10/22/2020	P / P	MB	App	90 days	499.80 €	–
Invirto	Agoraphobia, Panic disorder, Social phobias	CBT	12/03/2020	P / P	MB	App	90 days	620.00 € (incl. VR glasses)	–
elevida	Fatigue in multiple sclerosis	CBT	12/15/2020	F	MB	Browser	90 days	743.75 €	–
M-sense Migräne	Migraine	Headache diary, Prophylaxis exercises	12/16/2020	P / P	MB, PSVV	App	90 days	219.98 €	–
Selfapys Online-Kurs bei Depression	Depression	CBT	12/16/2020	P / P	MB	Browser	90 days	540,00 €	–
Rehappy	Stroke	Motivation, Education	12/29/2020	P / P	MB, PSVV	App, Browser	90 days	Initial prescription: 449,00 € (incl. energy band) Follow-up: 299.00 €	–
deprexis	Depression	CBT	02/20/2021	F	MB	Browser	90 days	297.50 €	–
Mika	Cancer	Symptom Documentation, Education	03/25/2021	P / P	MB	App	90 days	499.00 €	–
Mindable	Agoraphobia, Panic disorder	CBT	04/29/2021	P / P	MB, PSVV	App	90 days	576.00 €	–
CANKADO PRO-React Onco	Breast cancer	Symptom documentation	05/03/2021	P / P	PSVV	App, Browser	90 days	499.80 €	–
vorvida	Harmful alcohol Consumption, alcohol Dependence	CBT	05/06/2021	F	MB, PSVV	Browser	90 days	476.00 €	–
Selfapys Online-Kurs bei Generalisierter Angststörung	Generalized anxiety disorder	CBT	06/19/2021	P / P	MB	Browser	90 days	540.00 €	–
Selfapys Online-Kurs bei Panikstörung	Panic disorder	CBT	06/19/2021	P / P	MB	Browser	90 days	540.00 €	–
NichtraucherHelden-App	Tobacco dependence	CBT	07/03/2021	P / P	MB	App	90 days	Initial prescription: 239.00 € Follow-up: 99.00 €	–
ESYSTA App & Portal	Diabetes	Diabetes diary	07/04/2021	P / P	MB	App, Browser	90 days	249.86 €	–
Mawendo	Knee pain	Physiotherapy	08/09/2021	P / P	MB	Browser	One-time license	119.00 €	–
Oviva Direkt für Adipositas	Obesity	Multimodal Obesity therapy	10/03/2021	P / P	MB	App	90 days	345.00 €	–
companion patella	Knee pain	Physiotherapy	10/04/2021	P / P	MB	Browser	90 days	345.10 €	–
Novego	Depression	CBT	10/10/2021	P / P	MB	Browser	90 days	249.00 €	–
HelloBetter Stress und Burnout	Stress, burnout	CBT	10/18/2021	F	MB	Browser	90 days	599.00 €	–
HelloBetter Diabetes und Depression	Depression in diabetes mellitus	CBT	12/11/2021	F	MB	Browser	90 days	599.00 €	–
Kranus Edera	Impotence of organic origin	Physical training, Psychotherapy, mindfulness and sexual therapy	12/18/2021	P / P	MB, PSVV	App	90 days	656.88 €	–
HelloBetter ratiopharm chronischer Schmerz	Chronic pain	CBT	12/18/2021	P / P	MB	Browser	90 days	599.00 €	–
Cara Care für Reizdarm	Irritable bowel syndrome	CBT, dietary support	12/26/2021	P / P	MB, PSVV	App	90 days	718.20 €	–
HelloBetter Vaginismus Plus	Vaginismus	CBT	02/04/2022	F	MB	Browser	90 days	599.00 €	–
neolexon Aphasie	Aphasia, apraxia	Logopedic training	02/06/2022	P / P	MB	App, browser	90 days	487.90 €	–

CBT: cognitive behavioural therapy; MB: medical benefit; MP: freely-set manufacturer price; NP: negotiated price; pVE: positive Versorgungseffekte (positive healthcare effects); pSVV: patientenrelevante Struktur- und Verfahrensverbesserungen in der Versorgung (patient-relevant improvements of structure and processes); P: provisional; F: final

^a^For DiGA with provisional listing (i.e., whose study results are not yet available), study design and postulated benefit are stated as described in the DiGA directory.

^b^Represents the current freely-set manufacturer price or, if the manufacturer price has already been replaced by a negotiated price, the last valid manufacturer price before the negotiated price came into effect.

^c^Includes negotiated prices and prices set by the arbitration board, which replace the freely-set manufacturer prices by the 13th month after listing in the DiGA directory.

**Table 2:** Study design and characteristics of positive effects on healthcare of DiGA listed in the DiGA directory (as of 02/28/2022).

DiGA-Name	Indication(s)	Current listing status^a^	Study design^b^	Patient-relevant improvements of structure and processes ^a^	Medical benefit^a^
Kalmeda	Tinnitus	Final	RCT	–	tinnitus burden
velibra	Generalized anxiety disorder, Panic disorder (with/without agoraphobia), Social phobias	Final	RCT	General mental stress	anxiety symptoms, depression symptoms
somnio	Insomnia	Final	RCT	–	insomnia symptoms
Vivira	Back pain	Final	RCT	–	back pain
zanadio	Obesity	Provisional	RCT	–	body weight, body fat distribution, quality of life
Invirto	Agoraphobia, panic disorder, social phobias	Provisional	RCT	–	anxiety symptoms, quality of life
elevida	Fatigue in multiple sclerosis	Final	RCT	–	fatigue severity
M-sense Migräne	Migraine	Provisional	RCT	Health literacy, Self–efficacy	migraine burden, quality of life
Selfapys Online-Kurs bei Depression	Depression	Provisional	RCT	–	depression symptoms
Rehappy	Stroke	Provisional	RCT	Adherence, Activities of daily living, Health literacy, Self–efficacy	relapse rate, depression symptoms, quality of life
deprexis	Depression	Final	RCT	–	Depression symptoms
Mika	Cancer	Provisional	RCT	–	Quality of life
Mindable	Agoraphobia, panic disorder	Provisional	RCT	Health literacy, Anxiety–related control beliefs/ Self–efficacy, Activities of daily living	Anxiety symptoms, Panic symptoms, Quality of life
CANKADO PRO-React Onco	Breast cancer	Provisional	n/a	Health literacy	–
vorvida	Harmful alcohol consumption, Alcohol dependence	Final	RCT	Self–efficacy	Alcohol consumption
Selfapys Online-Kurs bei Generalisierter Angststörung	Generalized anxiety disorder	Provisional	RCT	–	Anxiety symptoms, Depression symptoms
Selfapys Online-Kurs bei Panikstörung	Panic disorder	Provisional	RCT	–	Anxiety symptoms, Depression symptoms
NichtraucherHelden-App	Tobacco dependence	Provisional	RCT	–	Smoking prevalence, Quality of life
ESYSTA App & Portal	Diabetes	Provisional	RCT	–	HbA1c
Mawendo	knee pain	Provisional	RCT	–	Knee pain, Functional state
Oviva Direkt für Adipositas	obesity	Provisional	RCT	–	Body weight
companion patella	Knee pain	Provisional	RCT	–	Knee pain, Functional state
Novego	Depression	Provisional	RCT	–	Depression symptoms
HelloBetter Stress und Burnout	Stress, burnout	Final	RCT	–	Stress level
HelloBetter Diabetes und Depression	Depression in diabetes mellitus	Final	RCT	–	Depression symptoms
Kranus Edera	Impotence of organic origin	Provisional	RCT	Self–management	Erectile function, Quality of life
HelloBetter ratiopharm chronischer Schmerz	Chronic pain	Provisional	RCT	–	Pain level
Cara Care für Reizdarm	Irritable bowel syndrome	Provisional	RCT	Work productivity/ Activities of daily living, health literacy	Irritable bowel symptoms, Anxiety symptoms, Depression symptoms, Quality of life
HelloBetter vaginismus plus	Vaginismus	Final	RCT	–	Vaginal penetration ability
neolexon aphasie	Aphasia, apraxia	Provisional	RCT	–	Linguistic skills

RCT: randomized controlled trial

^a^Finally listed DiGA have already proven their positive effect on healthcare. For provisionally listed DiGA, this evidence is currently still pending.

^b^For DiGA with provisional listing (i.e., whose study results are not yet available), study design and postulated benefit are stated as described in the DiGA directory.

## Pricing mechanisms for DiGA

The DiGA legislation provides different pricing mechanisms for the first twelve months and the period from the beginning of the 13th month after listing in the DiGA directory. During the first year after the inclusion of a DiGA in the directory, manufacturers are generally free to determine their sales price and pricing model. However, the framework agreement between the GKV-SV and the manufacturers’ umbrella organizations may specify maximum prices for comparable DiGA, limiting the amount of preliminary reimbursement by the GKV in a manner analogous to reference prices for drugs. As the GKV-SV and the manufacturers’ associations were unable to agree on a maximum price regulation, this was set by arbitration. The arbitration board has decided that the maximum prices will be calculated solely based on available freely-set manufacturer prices (and not on negotiated prices) within a group of comparable DiGA. The grouping will be based on the chapter structure of the ICD-10-GM.

From the beginning of the 13th month after the inclusion of a DiGA in the DiGA directory, the freely-set manufacturer price is replaced by a price agreed upon by the GKV-SV and the manufacturer in joint price negotiations. Such an agreement has a minimum validity of 12 months and may also include performance-related price components. If the negotiating parties do not reach an agreement within nine months after the inclusion of an application in the DiGA directory, the price shall be determined by arbitration within three months. For provisionally listed DiGA, the independent arbitration board determines the price if the negotiating parties are unable to come to an agreement within three months of the BfArM’s decision on the permanent inclusion of the DiGA in the directory. This takes place no later than three months after the end of the trial phase. Prices agreed or set after the beginning of the 13th month apply retroactively. The difference between the new negotiated price and the original freely-set manufacturer price must then be compensated for the period between the end of the first year and the date of the reimbursement agreement by repayment or subsequent reimbursement. In the framework agreement between the GKV-SV and the manufacturers’ umbrella organizations, threshold values for permanent reimbursement amounts can be defined. If the freely-set manufacturer price falls below such a threshold value, further price negotiations no longer need to take place. Like the maximum prices for the provisional reimbursement, the threshold regulations for the permanent reimbursement were the subject of arbitration proceedings, too. The arbitration board has decided that a freely-set manufacturer price must meet two conditions in order to not induce price negotiation: (1) The price must be below the limit of 25% of the average price of all DiGA included in the DiGA directory—taking into account the freely-set manufacturer’s prices as well as the negotiated prices, both of which standardized on a daily basis—and (2) the revenue within the last twelve months must not exceed 750,000 € including VAT. It is unclear which considerations were decisive for determining the level of the revenue threshold. However, with only six of the ten DiGA listed in Q4 2020 having exceeded €750,000 in sales by the end of Q3 2021, it is likely that this criterion is less restrictive than the price criterion, which is currently met by only one application (“Mawendo”; knee pain) [11]. Nevertheless, sales expectations may increase as DiGA become better known and have been on the market for a longer time, so that the revenue threshold will become more relevant in the future.

## Criteria for DiGA price negotiations

The documents submitted by the parties and the information published in the DiGA directory constitute the basis for the price negotiations. The manufacturer shall submit the following documents to the GKV-SV before the start of negotiations:

- The evidence on general requirements and pVE to the BfArM submitted as part of the fast-track process.
- The results of the studies conducted as part of the possible trial phase.
- Information on prices for self-payers.
- Information on prices in other European countries.
- The complete notification of the BfArM about the inclusion of the DiGA in the DiGA directory.
- The number of activation codes/prescription codes redeemed for the DiGA in the period from inclusion in the DiGA directory to five days before transmission.

In addition, the negotiating parties are entitled to provide the other negotiating party with “other price-relevant documents”. This may include, in particular, (economic) analyses of claims data or other real-world data that were collected after a DiGA was included in the DiGA directory, as well as studies on pVE that were completed after a DiGA was included in the DiGA directory.

Different provisions need to be highlighted concerning the reference points for the prices to be negotiated. Generally, Sect. 134 (4) Sentence 2 SGB V stipulates that the standards for the price negotiations must consider “whether and to what extent” evidence for pVE has been provided. This guideline is further specified in the framework agreement between the GKV-SV and the manufacturers’ umbrella organizations. According to Sect. 8 (2) Sentence 1 of the framework agreement, the reimbursement amounts are agreed “taking into account all price-relevant information resulting from the documents […] and in compliance with the statutory requirements in the individual case”. Furthermore, Sect. 8 (2) Sentence 2 of the framework agreement explicitly emphasizes the extent of the proven medical benefit and the extent of the proven pSVV as important pricing criteria to be considered. With the decision of the BfArM on the permanent inclusion in the DiGA list, the pVE (medical benefit and/or pSVV) are considered to be proven. The contents of the BfArM’s notification are binding for the negotiating parties.

## Discussion on DiGA prices

After more than one year of reimbursable DiGA in the German healthcare system, pricing remains an essential point of contention among stakeholders. Considering the high number of DiGA without conclusive evidence for their actual benefit, payers criticize the wide latitude afforded to manufacturers in setting prices in the first year after listing in the DiGA directory. In a recent position paper, the GKV-SV, therefore, advocates that the evidence requirements for DiGA, which it considers inadequate, should be gradually brought into line with the standards in other areas of healthcare [12]. In addition, the GKV-SV states that free pricing should be restricted to prevent unreasonably high manufacturer prices. Manufacturers’ associations counter the payers’ criticism by stating that a provisional listing in the DiGA directory also requires a plausible rationale for the potential pVE based on a preliminary data evaluation [13]. Furthermore, they argue that with the provisional listing in the DiGA directory, the legislature has deliberately opted for low-threshold access and trial options to promote innovation. Accordingly, the abolition of this regulation would be associated with a drastic reduction in the number of available DiGA.

Currently, freely-set manufacturer prices range from 119 € to 744 € per initial prescription (see Table 1). For all but one DiGA the price refers to a therapy cycle of 90 days. The price of “Mawendo” (knee pain; 119 €) refers to a one-time license for insured persons. Thus, it is the only application that would meet the price criterion of the threshold regulation described above. On average, the freely-set manufacturer price per initial prescriptions in the first year amounts to 456 €. Noteworthy, prices for permanently listed DiGA are higher than those for provisionally listed. At 532 €, their average price exceeds the average price of provisionally listed DiGA, which is 429 €, by 24% (median: 493 € vs. 538 €; +9%). Although this could suggest that differences in the available evidence are already included in the manufacturer pricing, it must be taken into account that the corresponding price differences could also be influenced by several other factors (e.g., the size of the patient population addressed or strategic considerations by the manufacturers).

Recently, the discussion about appropriate DiGA prices has intensified in view of the market entry of numerous applications with above-average prices and repeated price increases by manufacturers in the first year after market access. Thus, the 90-day prices of the six DiGA listed since December 2021 are on average 34% higher than the current average price within the first year. Furthermore, in January 2022 the first permanent price for a DiGA (“somnio”; insomnia) has become available. As the negotiating parties were unable to agree on a compromise, the arbitration board set a price of 225 € for a prescription period of 90 days applicable from the 13th month after market access. This is around 52% lower than the previous price of the first year (464 €), and once again raised concerns about the appropriateness of the freely set manufacturer prices at market access.

## Implementing value-based pricing for DiGA

Considering the drawbacks of other frequently discussed pricing approaches (e.g., cost-based pricing), it is to be welcomed that the German legislature has moved patient-relevant benefits into the focus of DiGA pricing [14–16]. However, neither the SGB V nor the framework agreement between the GKV-SV and the manufacturer’s associations provide clear guidelines or an algorithm on how a reasonable, value-based DiGA price can and should be derived. Furthermore, in its listing decision, the BfArM only makes a binary statement about whether a DiGA has a pVE, but not about the extent of this effect. In contrast, the early benefit assessment for new pharmaceuticals (the so-called AMNOG procedure), whose processes are quite similar to the DiGA fast-track process, examines the extent of the additional benefit compared to a comparative therapy. The official decision on the extent (differentiated according to six categories) is then directly considered in the price negotiations between the pharmaceutical manufacturer and the GKV-SV.

This lack of a binding decision on the extent of the pVE in relation to a clearly defined comparative therapy poses a challenge for DiGA price negotiations and creates a scope of discretion in implementing value-based pricing [17]. However, to meet legal requirements and the goal of a fair balance of interests in negotiations, these should be based on transparent methods for translating patient-relevant benefits into reasonable prices. In principle, various approaches can be considered for the value-based pricing of DiGA [18]. Specifically focusing on the area of digital health, Powell and Torous recently proposed an “A Patient-Centered Framework for Measuring the Economic Value of the Clinical Benefits of Digital Health Apps” [19]. They present a pragmatic, QALY-based model to guide informed decision-making around app pricing. To calculate the economic value of the clinical benefits of digital health apps, the model considers the (country-specific) monetary value of a QALY; the estimated QALYs lost due to the disease addressed by the app, the average effect size of the app’s health impact, the engagement rate of app users as well as the duration of the app’s impact.

Generally, this approach could also be applied in DiGA price negotiations and provide a benchmark for an appropriate, value-based price. However, the establishment of QALY-based methods in Germany has proven difficult, especially due to ethical reservations. Consequently, as shown in Table 2, pVE of the DiGA listed to date are mostly represented by indication-specific (e.g., disease-specific symptoms) instead of universal outcome measures such as QALYs. As studies of societal willingness to pay for such specific measures (and thus outcome-specific cost-effectiveness thresholds) are usually not available, an approach to value-based pricing must include considerations about what might be judged an appropriate relation of costs and benefits of a DiGA. Considering these circumstances, the Advisory Council on the Assessment of Developments in the Health Care System (“Sachverständigenrat zur Begutachtung der Entwicklung im Gesundheitswesen”, SVR Gesundheit), which advises the German Ministry of Health, suggests that DiGA prices should be evaluated in light of the cost-effectiveness ratios of DiGA and in comparison to existing services in SHI care [20].

**Fig. 2:**
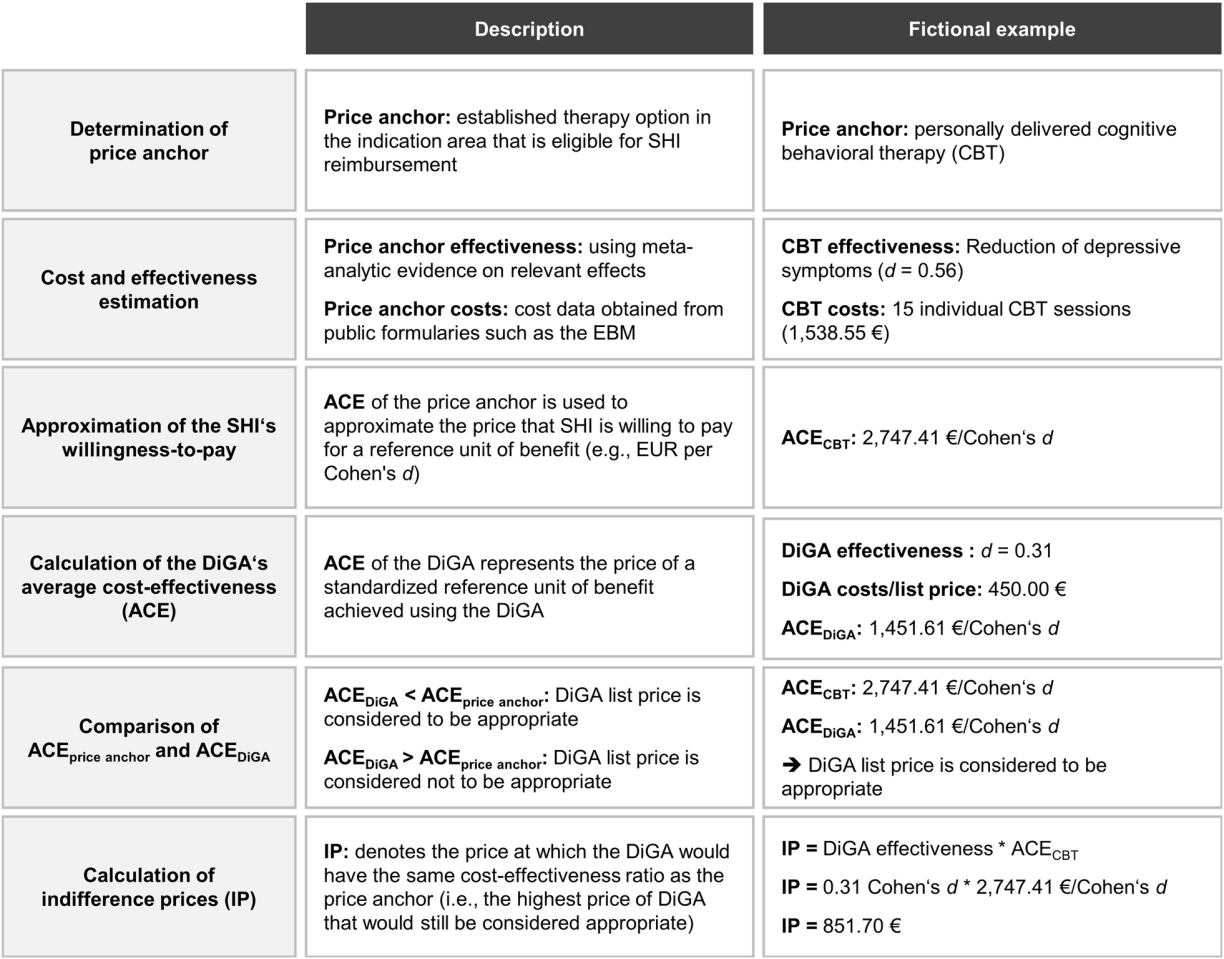
Assessing the appropriateness of the price of a cognitive-behavioral therapy-based DiGA for symptom reduction in depression (fictional example; CBT cost data based on EBM)

Following this proposal, value-based DiGA prices could pragmatically be derived from the observed effects of a DiGA and the cost-effectiveness of other therapies in the addressed indication that SHI already reimburses. A corresponding approach is presented hereafter. Essentially, it is based on a similar normative assumption as the efficiency frontier concept proposed by the German Institute for Quality and Efficiency in Health Care (“Institut für Qualität und Wirtschaftlichkeit im Gesundheitswesen”, IQWiG) [21]. Thus, in the absence of consensus willingness-to-pay thresholds for indication-specific outcome measures, the observed cost-effectiveness of established therapies is considered the best available proxy for the minimum collective willingness-to-pay of SHI insurants. Accordingly, a price would be judged (at least) appropriate if a unit of benefit generated by the DiGA costs less than or at most the same as a unit of benefit generated by the existing therapy. Although health economic evaluations typically make use of incremental cost-effectiveness ratios, such an incremental approach is likely to be not feasible in the DiGA setting, as German health authorities are fundamentally opposed to the application of general cost-effectiveness thresholds. Moreover, the alternative derivation of an appropriate incremental cost-effectiveness ratio by extrapolation of the last segment of the efficiency frontier would require consideration of all relevant therapeutic options in the therapeutic area and would thus be very time-consuming. Therefore, we propose an approach that bases the assessment of the appropriateness of a DiGA price on a comparison of the average cost-effectiveness of a DiGA and a comparator therapy used as a price anchor (each related to its non-use). The approach is schematically shown in Fig. 2:

### Determination of a therapy modality as a price anchor

The first step is to select a therapy modality that serves as a benchmark or price anchor for a reasonable, value-based price. On the one hand, this decision should be based on the guideline recommendations in the indication area and the congruence of therapy goals between the price anchor and the DiGA. On the other hand, it must also consider factors such as the availability of evidence on the effectiveness and price parameters. In addition, the therapy that serves as the price anchor must also be eligible for reimbursement by the SHI. Thus, personally delivered cognitive behavioral therapy might be an appropriate price anchor for a DiGA that supports depression therapy in order to reduce depression symptoms (see Fig. 2). It should be noted, however, that in principle more than one therapy modality can be taken into account as a price anchor, if this is suggested by the guideline recommendations, the reality of care in SHI, and the available evidence on the benefit of different therapy modalities.

### Cost and effectiveness estimation

The second step is to determine cost and effectiveness parameters for the price anchor. Costs for SHI services can regularly be obtained from public formularies such as the uniform value scale (“Einheitlicher Bewertungsmaßstab”, EBM) for outpatient services. When obtaining effectiveness parameters, the classic hierarchy of evidence should be considered. Thus, meta-analyses of RCT are the preferred standard of evidence. The effectiveness parameters determined for the price anchor must be congruent with the pVE of the DiGA under consideration (e.g., reduction of depression symptoms). Common standardized effect measures such as Cohen’s *d* are suitable as reference unit of benefit. However, it should be noted that the observed effectiveness may depend on various factors, such as the comparator therapy used in the trials or the duration of therapy. In determining appropriate effect estimates for the price anchor, it has to be considered that DiGA studies usually compare the use of a DiGA with its non-application – and not, for example, with a single therapeutic standard. A similar comparison should also be decisive for the effect sizes considered for the price anchor. In addition, costs also depend on the assumed duration and scope of the therapy. Therefore, it is important to make evidence-based assumptions and to choose a duration of therapy that on the one hand corresponds to the reality of care in SHI and on the other hand ensures the achievement of the assumed effects with a sufficient probability. To account for uncertainty in both the cost and effectiveness parameters of the price anchor, ranges (e.g., based on confidence intervals) can be considered instead of individual estimators.

### Approximation of the established minimum willingness to pay

In the third step, the determined cost and effectiveness parameters of the price anchor are put in relation to each other to obtain the average cost-effectiveness of the price anchor. Depending on whether one or more cost and/or effectiveness estimators are included, one or more cost-effectiveness ratios may result for the price anchor. Each of these cost-effectiveness ratios reflects an estimate of the price that SHI currently pays for a reference unit of benefit (e.g., € per Cohen’s *d*) based on the treatment modality specified as the price anchor – and thus an approximation of the collective minimum willingness-to-pay of SHI insurants for a respective benefit.

### Calculation of the cost-effectiveness ratio of the DiGA

In the fourth step, the average cost-effectiveness for the DiGA is determined under consideration of the available evidence and list prices. To account for any uncertainties in the body of evidence, more than one cost and effectiveness parameter can be used in this case as well. This can be done, for example, by including the upper and lower bounds of the confidence interval of the effect estimator as effectiveness parameters or considering single and multiple prescriptions for the cost calculation.

### Comparison of cost-effectiveness ratios and calculation of indifference prices

In the fifth and final step, the given DiGA price is examined for appropriateness. For this purpose, the average cost-effectiveness of the DiGA and the price anchor can be compared, and it can be analyzed whether (or in how many cases) the DiGA has a better cost-effectiveness ratio than the price anchor. For a more detailed analysis of a reasonable price, indifference prices of DiGA can be calculated from its observed effect sizes and the cost-effectiveness ratios of the price anchor through simple multiplication (DiGA effect size * price anchor cost-effectiveness ratio). For each combination of DiGA effect size and price anchor cost-effectiveness ratio, this indifference price indicates the price at which the DiGA would just have the same cost-effectiveness ratio as the price anchor. If the indifference price is higher than or equal to the list price of the DiGA, the list price may be considered reasonable given the pVE of the DiGA. To account for the distribution of indifference prices, it is useful to look at statistical measures of location such as the mean and median.

## Conclusions

Making DiGA available to all insured persons in Germany represents a significant step toward comprehensive digital healthcare. The legislature has established a legal framework for structured market access and pricing of DiGA. At the core of these regulations is a fast-track application and evaluation procedure, in which a DiGA’s patient-relevant benefits to the health of patients as well as to care structures and processes are evaluated. The result of the evaluation by the BfArM is a binary statement about whether or not such pVE exist. After a year of freely-set manufacturer prices, DiGA manufacturers negotiate prices with the GKV-SV. Yet, the legislature as well as the contracting parties have only created a general framework for these price negotiations. This framework emphasizes the pVE as a central criterion for pricing. However, the legislature does not provide any further guidance on the implementation of value-based pricing.

Just over a year after DiGA were introduced into the SHI system, 30 DiGA have already become eligible for reimbursement. The DiGA market is thus growing by approximately two new applications per month and already covers a wide range of indications. However, especially in the context of the relatively low evidence requirements for listing in the DiGA directory and the possibility of free pricing in the first year, the pricing of DiGA is a central point of contention. While health insurers urge for a restriction on free pricing and an increase in evidence standards, manufacturers and their associations emphasize that the current system design is fundamental to the successful development of the digital care sector. To what extent the freely-set manufacturer prices already consider factors such as the (expected) benefit of a DiGA cannot yet be assessed based on the available data. In particular, it will need to wait until more study results on the numerous provisionally listed DiGA and their negotiated prices are available.

With regard to the price negotiations, it is to be welcomed that the legislature and the contracting parties to the framework agreement have made the patient-relevant benefit of a DiGA a central price assessment criterion. However, the extent of a DiGA’s pVE is not determined in the context of BfArM’s decision, leaving the translation of the patient-relevant benefit into a value-based price largely up to the negotiating parties. Yet, the practical implementation of value-based pricing has to consider some peculiarities resulting from the regulatory framework. For example, most DiGA are not evaluated versus a single standard of care and the studies usually consider indication-specific outcome measures, making common methods of health economic evaluation such as an incremental analysis and the cross-indication application of cost-effectiveness thresholds difficult to implement. Furthermore, the tight time frame in which the price negotiations take place must be taken into account. Given this setting, we provided a pragmatic approach to assess the appropriateness of DiGA prices that grounds on the effectiveness of a DiGA and the SHI’s established willingness to pay approximated by the average cost-effectiveness of other reimbursable services in the therapeutic area. This evidence-based approach is easy to apply and allows a basic assessment of the cost-effectiveness ratio of a DiGA and thus the appropriateness of its list price.

Limitations of this approach need to be considered. Due to its clear relatedness, widely-discussed points of criticism of the IQWiG’s efficiency frontier concept also apply here [22–24]. These especially include that an indication-specific assessment of cost-effectiveness may lead to inconsistent decisions across therapeutic areas and diseases and thus allow for inefficiencies [22]. Moreover, critics emphasize that anchoring the prices of new technologies to the observed cost-effectiveness of existing technologies which are usually not priced in terms of their value also implies inefficiencies [24]. We acknowledge and share these concerns. However, value-based pricing for DiGA has to operate within the regulatory framework described above. Our approach aims to guide price negotiations (rather than reimbursement decisions) with these specifics in mind. It provides an objective benchmark for how much a DiGA may cost without increasing the price of a unit of benefit already paid in a therapeutic area. Nevertheless, the concerns about the appropriateness of regulatory requirements from a health economics perspective and from a system-level efficiency perspective remain and need to be further discussed in the future.

Furthermore, although pVE were put in focus of DiGA pricing, economic analyses are no official component of the negotiation process. Thus, supporting analyses have to fit into the existing procedure and their time frame. However, the construction of an efficiency frontier that covers all relevant therapy options in a therapeutic area is likely to be an “difficult, tedious, and time- and resource-consuming activity.“ [24] For this and other reasons (e.g., non-acceptance of ICER approach by German health authorities), we based our approach on the average cost-effectiveness of one (or more) price anchor. Thus, although an incremental approach, by its very nature, allows for more accurate conclusions about the consequences of introducing a new technology, we consider the use of the average cost-effectiveness to be appropriate (albeit not optimal) in the current DiGA context. This applies in particular as it is typically more restrictive with regard to assessing the appropriateness of a price, resulting in rather conservative estimates [23].

In addition to these general methodological aspects, practical implementation problems may arise. The selection of the price anchor as well as cost and effectiveness parameters can pose a difficulty. As long as there are no legal requirements in this regard, the determination will not be made by the BfArM or another external institution, but must also be integrated into the deliberative negotiation process. This requires a willingness to cooperate and reach a consensus on the side of the negotiating partners. In particular, when the evidence base is uncertain and assumptions must be made, this offers further potential for conflict. Generally, such uncertainties should be addressed transparently by employing deterministic and probabilistic sensitivity analyses as also used in classic cost-effectiveness analysis. An a example for a probabilistic derivation of the efficiency frontier is provided by Mühlbacher and Sadler [25].

Furthermore, the existence of multiple pVE for the same DiGA can pose a challenge. For an appropriate consideration, the different effects would need to be considered in value-based pricing according to their relevance. However, this would again require evidence on patient preferences, e.g., from DCE studies [25]. In addition, our approach is only applicable when adequate evidence based on the endpoints relevant for the DiGA is also available for the selected price anchor. Thus, if the pVE is primarily based on structural effects (i.e., pSVV), its application is likely to be difficult. Currently, only one in 30 DiGA bases its pVE solely on structural effects. However, in future there might also be DiGA that, for example, primarily address medication adherence. If medical benefits are not considered in addition to adherence parameters alone, the approach we have outlined is likely to reach its limits. In this case, it would be up to the negotiating parties to find a way to translate pSVV into an appropriate price. Less problematic, on the other hand, are cases with pSVV in addition to a medical benefit. The pSVV could then be used as a possible supportive argument in the negotiations to also allow the DiGA to have an average cost-effectiveness (based on the medical benefit) that is worse than that of the price anchor.

This illustrates that our approach to assessing an appropriate DiGA price is only one part of the pricing puzzle. In the absence of further guidance on the implementation of value-based pricing, it can provide a benchmark for price negotiations between GKV-SV and manufacturers. However, these negotiations also have to account for possible benefits not incorporated in the cost-effectiveness estimations as well as other information considered “price-relevant”. The latter may include prices for self-payers or in other European countries as mentioned in the framework agreement as well as other factors as the number of therapeutic options in the field of indication, the size of the addressed patient population, and, not least, the expected budget impact.

## Data Availability

All data generated or analyzed during this study are included in this published article.

## Abbreviations

BfArM: Federal Institute for Drugs and Medical Devices (Bundesinstitut für Arzneimittel und Medizinprodukte)
DiGA: Digital health applications (Digitale Gesundheitsanwendungen)
DiGAV: Digital Health Applications Regulation (Digitale Gesundheitsanwendungen-Verordnung)
DVG: Digital Healthcare Act (Digitale-Versorgung-Gesetz)
EBM: Uniform value scale (Einheitlicher Bewertungsmaßstab)
GKV-SV: National Association of Statutory Health Insurance Funds (GKV-Spitzenverband)
MDD: Medical Device Directive
MDR: Medical Device Regulation
pSVV: Patient-relevant improvements of structure and processes (patientenrelevante Struktur- und Verfahrensverbesserungen)
pVE: Positive healthcare effects (positive Versorgungseffekte)
RCT: Randomized controlled trial
SHI: Statutory health insurance
SVR Gesundheit: Advisory Council on the Assessment of Developments in the Health Care System (Sachverständigenrat zur Begutachtung der Entwicklung im Gesundheitswesen)
